# ADOLESCENT STUDENTS' ATTITUDES TOWARDS SEXUALITY: THE CONSTRUCTION AND VALIDATION OF A SCALE

**DOI:** 10.1590/1984-0462/2021/39/2019372

**Published:** 2020-11-16

**Authors:** Teresa Madalena Kraus Brincheiro Hüttel Barros, Sónia Isabel Horta Salvo Moreira de Almeida Ramalho, Clementina Maria Gomes de Oliveira Gordo, João Manuel Graça Frade, Alexandra Luz, Pascoal Moleiro, Maria dos Anjos Coelho Rodrigues Dixe

**Affiliations:** a Center for Innovative Care and Health Technology (ciTechCare), Leiria, Portugal.; b Instituto Politécnico de Leiria, Leiria, Portugal.; c Centro Hospitalar de Leiria, Leiria, Portugal.

**Keywords:** Attitude, Teenagers, Sexuality, School, Atitude, Adolescentes, Sexualidade, Escola

## Abstract

**Objective:**

To construct a scale of adolescents' attitudes towards sexuality and to determine their psychometric characteristics.

**Methods:**

Methodological study conducted with 394 students from the 8th to 12th grades of a school in central Portugal. They answered a questionnaire consisting of the Adolescent Students' Attitude Scale for Sexuality (E3AS) and socio-demographic and academic data. The project received a favorable opinion from the National Data Protection Commission (authorization No. 10421/2017). Construct validity analysis was performed through exploratory factor analysis and internal consistency was performed through Cronbach's alpha. A maximum error probability of 5% was considered.

**Results:**

The mean age of the sample was 14.9±1.4 years, with 53.3% being female. The instrument consisted of 34 items distributed into five factors: F1. Family planning and sex education (α=0.826); F2. First sexual relationship (α=0.819); F3. Violation of sexual rights and who to turn to in the event of unplanned pregnancies (α=0.695); F4. Gender expression and identity (α=0.542), and F5. Unplanned pregnancy and parenting (α= 0.761), with a total alpha value of 0.766, accounting for 45.3% of total variance.

**Conclusions:**

The psychometric adequacy of the E3AS for the Portuguese population allows us to affirm that it is a valid and reliable instrument that can be used in future studies to assess the attitudes of adolescent students towards sexuality.

## RESUMO

**Objetivo:**

Construir uma escala de atitudes dos adolescentes em face da sexualidade e determinar as suas caraterísticas psicométricas.

**Métodos:**

Estudo metodológico realizado com 394 alunos do 8º ao 12º ano de escolaridade de uma escola da região Centro de Portugal que responderam a um questionário constituído da Escala de Atitudes dos Alunos Adolescentes em face da Sexualidade e de dados sociodemográficos e acadêmicos. O projeto recebeu parecer favorável da Comissão Nacional de Proteção de Dados (autorização nº 10421/2017). Realizou-se a análise de validade do constructo, por análise fatorial exploratória, e de consistência interna, por alfa de Cronbach. Foi considerada probabilidade de erro máximo de 5%.

**Resultados:**

A média de idades da amostra foi de 14,9±1,4 anos, e 53,3% dos participantes eram do sexo feminino. O instrumento foi constituído de 34 itens distribuídos por cinco fatores: Fator 1. Planejamento familiar e educação sexual (α=0,826); Fator 2. Primeira relação sexual (α=0,819); Fator 3. Violação dos direitos sexuais e a quem recorrer na gravidez não planejada (α=0,695); Fator 4. Expressão e identidade de gênero (α=0,542); Fator 5. Gravidez não planejada e parentalidade (α=0,761). O valor do alfa total da escala foi 0,766, o que explica 45,3% da variância total.

**Conclusões:**

A adequação psicométrica da escala para a população portuguesa permite afirmar que se trata de um instrumento válido e confiável, que poderá ser utilizado em estudos futuros com o intuito de avaliar as atitudes dos alunos adolescentes em face da sexualidade.

## INTRODUCTION

The Europe 2020 Strategy,^1^ with regard to sustainable growth and inclusive education, and the United Nations 2030 Agenda for Sustainable Development, Promotion and Education for Health in schools have a relevant role in the “(…) development of healthy, sustainable and happy citizens and societies, which is why they contribute to the goals and objectives defined by the World Health Organization for Health and Wellbeing in Europe - Health 2020”.^2^

Schools assume a role par excellence in the mediation of previous and formal knowledge at all levels and cycles of education and teaching. The development of skills, combined with the promotion of values, attitudes and behaviors, allow adolescents to critically understand and intentionally participate in building a more just, inclusive and supportive reality, in the face of current challenges.^3^

The notion of freedom and responsibility emerges, when it is associated with choosing values, and it is translated by the definition of strategies for a dialogical attitude and social construction.^4^ Effective intervention requires approaching new themes, such as values, objectives, freedom with responsibility, and knowledge, that allow adolescents to be “free, autonomous, responsible and aware of themselves and the world that surrounds them” in order to make informed decisions.^5^

It is necessary to make adolescents more responsible with regard to reproduction, and to educate them on promoting human relations with respect for their own freedom, other's freedom, responsibility, coherence, equal rights and pleasure, listening and understanding of others.^5^ ^,^ ^6^

The growing interest in evaluating adolescents' attitudes towards sexuality leads to the need to build and validate a specific instrument for this population. As such, this methodological study aimed to build a scale of adolescents' attitudes towards sexuality, determine the psychometric characteristics of this scale and evaluate the relationship between adolescents' attitudes towards sexuality and some sociodemographic variables (age and sex).

## METHOD

The target population of this study was adolescent students from Portugal, and the sample consisted of adolescents who attended elementary and high school at two educational institutions in the central region of the country.^7^ The inclusion criteria were defined as: being a student of the 8th, 9th, 10th, 11th or 12th grade, of both sexes, having a command of the Portuguese language and being 19 years old or younger. Recruitment was carried out by the teachers of the schools, with interest expressed by the parents and approval of the students, after a presentation and awareness meeting for the project.

The data collection instrument was composed of two groups: sociodemographic and sexual characterization data and Adolescent Students' Attitudes Scale towards Sexuality (*Escala de Atitudes dos Alunos Adolescentes em face da Sexualidade* - E3AS). Although there are some scales to assess adolescents' attitudes towards sexuality, none included their various aspects. Thus, it was decided to build and validate one that included the following areas: Family planning and sex education; First sexual relationship; Violation of sexual rights and who to turn to in the event of an unplanned pregnancy; Gender expression and identity; Unplanned pregnancy and parenting. To develop it, we used guidelines from the World Health Organization (WHO) and the General Directorate of Health and Education, logotherapeutic principles, the development of Competence for Proactive Unconditional Care,^8^ a literature review, and meetings with health professionals.

The first version consisted of 41 items with a Likert-type answer, with five options: I totally disagree; I disagree; I do not agree nor disagree; I agree; and I totally agree. This first version of the instrument was subjected to review by health professionals (nurses and pediatricians) through a focus group. A thinking aloud moment and a pre-test were also carried out with 20 students who met the inclusion criteria defined for this study. No changes were proposed. The psychometric characteristics of the instrument will be presented in the results chapter.

All participants and their parents or guardians were previously informed of the objectives and purposes of the study, and were asked for informed consent, clarified in writing. In order to maintain the anonymity of the responses, a code was placed on each of the instruments.

Data were collected between April 23 and May 4, 2018, anonymously and confidentially. The students responded to the instrument in the computer room using Google Docs *.* The protocol was approved by the National Data Protection Commission (authorization No. 10421/2017 of 09/12/2017).

Descriptive statistics were used to characterize the sample, namely frequencies, measures of central tendency and variability. The internal consistency of the scale was calculated using Cronbach's alpha. The behavior of each item was verified using Pearson's correlation, and by Cronbach's alpha if the item was excluded. For the study, α> 0.6 was considered acceptable, α> 0.7 was good, α> 0.8 was very good and α> 0.9 was excellent.^9^

For each aspect, the construct validity was calculated using a factor analysis with a Varimax rotation. The Kaiser-Meyer-Olkin (KMO) test was used to assess the adequacy of exploratory factor analysis, in which a KMO value> 0.8 was considered to be good. To determine the factors, an *eigenvalue*> 1.0 was considered. The criteria for retaining items/factors in the exploratory factor analysis were: (a) saturation above 0.30, (b) non-inclusion of items that saturate two or more factors, with a difference of less than 0.10 between them, and (c) at least two items in each factor obtained.

Homogeneity indices (corrected total correlation) of the items were analyzed to determine whether it would be necessary to eliminate some of them.

Pearson's correlation was used to study the relationships between the variables. The choice for parametric tests was due to the fact that, in each group, the sample was greater than 30, thus we opted to apply the central limit theorem.^10^

Through the descriptive analysis, a construct validity analysis was performed by means of a factor analysis. Internal consistence was analyzed by calculating Cronbach's α (0.766). A maximum probability of error of 5% was considered.

## RESULTS

The sample consisted of 394 adolescents (mean of 14.9±1.4 years), of which 53.3% were male. The years of schooling varied between the 8th and 12^th^ grades, with the group of students who were in the 9th grade being the most representative (44.4%).

The psychometric characteristics of E3AS*,* were evaluated, namely its construct validity and internal consistency (reliability).

For the construct validity, starting from an initial scale consisting of 41 items, through several successive analyzes, the items with factorial loads below 0 were eliminated, obtaining a final version with 34 items grouped into five factors with eigenvalues greater than 1, which explained 45.30% of the total variance. Some items carried values greater than 0.3 in more than one factor. However, as the difference between the factor weights was greater than 0.15, they were not eliminated. All factors included in this solution were comprised of more than two items, and communalities had values greater than 0.3 and carried different factors with factor weights between 0.321 and 0.846. The sample adequacy index was 0.785, with p=0.0001, in such a way that the data matrix was considered adequate for factor analysis (Table 1).

**Table 1:** Factor analysis and weight of factors after Varimax of the Adolescent Students' Attitudes Scale towards Sexuality.

Item	communality	F1	F2	F3	F4	F5
23.	0.428				0.669	
24.	0.517				0.753	
25.	0.621				0.781	
26.	0.309				0.454	
27.	0.355				0.550	
28.	0.505				0.762	
29.	0.337				0.571	
30.	0.502				0.699	
31.	0.535				0.658	
32.	0.525				0.615	
11.	0.422		0.616			
12.	0.510		0.710			
13.	0.752		0.846			
14.	0.760		0.846			
15.	0.534		0.717			
16.	0.474			0.730		
17.	0.407			0.770		
18.	0.368			0.406		
19	0.379			0.574		
20.	0.324			0.321		
21.	0.358			0.488		
22.	0.384			0.438		
33.	0.589					0.759
34.	0.581					0.738
1.	0.467	0.507				
2.	0.529	0.632				
3.	0.546	0.671				
4.	0.582	0.523				
5.	0.344	0.405				
6.	0.504	0.720				
7.	0.582	0.736				
8.	0.518	0.714				
9.	0.341	0.622				
10.	0.247	0.432				
(eigenvalues)		5.92	3.22	2.35	2.13	1.75
% Var. explained		17.4%	9.5%	6.9%	6.3%	5.2%
% Var. accumulated		17.42%	26.9%	33.8%	40.1%	45.3%

F1: Family planning and sex education; F2: First sexual relationship; F3: Violation of sexual rights and who to turn to in the event of an unplanned pregnancy; F4: Gender expression and identity; F5: Unplanned pregnancy and parenting. Var.: variance; Kaiser-Mayer-Olkin = 0.785; Bartlett's sphericity test (Approx. chi-square=4773.101; p = 0.0001).

Factor 1 (F1), which saturated with items 1 to 10, was called Family planning and sex education; Factor 2 (F2), with items 11 to 15 was called First sexual relationship; Factor 3 (F3), with items 16 to 22 was called Violation of sexual rights and who to turn to in the event of an unplanned pregnancy; Factor 4 (F4), with items 23 to 32, was called Gender expression and identity; and Factor 5 (F5), with items 33 and 34 was called Unplanned pregnancy and parenting.

The E3AS reliability study was comprised by determining the internal consistency of the scale items. According to what is presented in Table 2, a global value of Cronbach's alpha of 0.766 and corrected correlation values ranging between 0.223 and 0.680 were obtained, data that confirm the internal consistency of the scale. It is important to note that some of the items have a Cronbach's alpha value that is slightly higher than the global alpha, however they were not excluded because their content is important and their exclusion does not improve the global scale value. Good Cronbach alphas were also obtained for each of the scale factors, as shown in Table 3. Pearson's correlation index between the various factors and the total scale was also calculated, the results of which are shown in Table 3.

**Table 2:** Homogeneity statistics of items and coefficients of internal consistency (**α**) of the Adolescent Students' Attitude Scale towards Sexuality.

	Average	SD	r corrected	Cronbach's α corrected
**F1: Family planning and sex education**				0.826
For you, does family planning mean:				
1. The prevention of sexually transmitted infections	3.8	0.8	0.308	0.758
2. Help having a healthy sex life	4.1	0.7	0.352	0.757
3. Information/obtaining contraceptive methods	4.1	0.7	0.425	0.754
4. Do you agree that both members of the couple should share the responsibility for family planning?	4.4	0.6	0.314	0.759
5. Do you consider it important to have places where you can talk to professionals about your doubts and problems related to sexuality?	4.3	0.6	0.361	0.757
In your opinion, sex education:				
6. Helps you to live your sexuality more responsibly	4.1	0.6	0.426	0.756
7. Helps you to have more information	4.2	0.5	0.464	0.755
8. Clarifies your doubts	4.2	0.8	0.360	0.758
9. Helps you not get sexually transmitted infections	3.9	0.7	0.287	0.759
10. Should be addressed at school	4.2	0.8	0.280	0.760
**F2 First sexual relationship**				0.819
Adolescents/young people have their first sexual relationship because:				
11. They drank too much	2.9	0.9	0.223	0.762
12. They got an older boyfriend or girlfriend	2.6	0.9	0.288	0.764
13. They are afraid that their partner will be angry	2.6	1.0	0.392	0.753
14. They are afraid that their partner will abandon them	2.7	1.0	0.348	0.756
15. They feel pressured by colleagues/friends	2.7	1.0	0.252	0.761
**F3. Violation of sexual rights and who to turn to in the event of an unplanned pregnancy**				0.695
In a couple, if one of the members tries to pressure the other into having sex and the other does not want it, they should:				
16. Refuse	4.4	0.6	0.461	0.753
17. Clearly say they are not interested	4.4	0.6	0.398	0.756
18. Do it so as not to lose them	4.3	0.8	0.297	0.759
19. Ask for respect of their decision	4.5	0.6	0.410	0.755
20. End the relationship	2.2	1.0	0.258	0.761
21. Ask their parents for help	4.4	0.5	0.337	0.759
22. Ask a healthcare professional for help	4.4	0.5	0.363	0.758
**F4. Gender expression and identity**				0.542
To what extent do you believe that:				
23. Genitals organs define someone as a man or woman?	2.8	1.3	0.297	0.759
24. Clothing defines someone as a man or woman	3.9	0.9	0.355	0.755
25. The use of makeup defines someone as a man or woman	3.9	1.0	0.406	0.752
26. Society defines the correct behavior for being a man or a woman	3.4	1.2	0.560	0.773
27. Each person must identify and accept himself or herself as a man or woman regardless of the sexual organs they have at birth	3.9	1.0	0.297	0.759
In adolescence, do you believe that masturbation:				
28. Is an act that should be avoided	2.2	0.9	0.229	0.767
29. Is a form of knowing one's body	1.9	0.8	0.333	0.773
30. Is mandatory	1.8	0.9	0.650	0.770
31. Is a behavior that harms health	1.9	0.8	0.298	0.768
32. I do not know what it means	1.7	0.9	0.229	0.767
**F5 Unplanned pregnancy and parenting**				0.761
33. If in a couple the woman becomes pregnant, they should have an abortion	3.8	0.9	0.680	0.770
34. If in a couple the woman becomes pregnant, they should keep the child	4.2	0.8	0.232	0.762
total Cronbach α				0.766

**Table 3:** Pearson's correlation between the factors and the total of the Adolescent Students' Attitudes Scale towards Sexuality.

		F1	F2	F3	F4	F5	Total
F1	Family planning and sex education	1					
F2	First sexual relationship	0.094	1				
F3	Violation of sexual rights, and who to turn to in the event of an unplanned pregnancy	0.470**	0.109*	1			
F4	Gender expression and identity	0.144**	0.067	0.275**	1		
F5	Unplanned pregnancy and parenting	0.025	0.013	0.149**	0.102*	1	
TOTAL	Total scale	0.678**	0.485**	0.685**	0.614**	0.256**	1

*The correlation is significant at the 0.05 level; **the correlation is significant at the 0.01 level.

Regarding the relationship between the total and the scale factors with sex and age, the parametric analysis (Table 4) revealed significant differences in the different factors and in the total scale of the adolescents' attitudes: sexuality at school, between the two sexes (p <0.05), with the exception of F5, whose variation was not different between males and females (p = 0.491). In general, females presented better results than males. As for the effect of age, only differences were found in F5, in which older adolescents had worse results than younger ones (p = 0.009).

**Table 4:** Variation of each of the factors and the total of the Scale of Attitudes of Adolescent Students towards Sexuality with sex and age.

Sex		n	Average	Standard deviation	p-value (sex)	p-value (age)
F1	Female	210	42.2	4.0	0.001**	0.622
	Male	184	40.7	4.7		
F2	Female	210	14.2	4.0	0.024*	0.311
	Male	184	13.4	3.5		
F3	Female	210	30.0	2.7	<0.001**	0.171
	Male	184	27.7	3.0		
F4	Female	210	27.6	3.5	<0.001**	0.154
	Male	184	24.6	4.4		
F5	Female	210	7.9	1.5	0.491	0.009*
	Male	184	8.1	1.6		
TOTAL	Female	210	122.1	8.8	<0.001**	0.226
	Male	184	114.8	9.9		

*The correlation is significant at the 0.05 level; **the correlation is significant at the 0.01 level.

## DISCUSSION

The results indicate that the instrument consisted of 34 items distributed by five factors: F1. Family planning and sex education (α=0.826); F2. First sexual relaitonship (α = 0.819); F3. Violation of sexual rights and who to turn to in the event of an unplanned pregnancy (α = 0.695); F4. Gender expression and identity (α = 0.542); and F5. Unplanned pregnancy and parenting (α=0.761). The total alpha value of the scale was 0.766, which explains 45.3% of the total variance. Although the average age of the sample in the present study (14.9±1.4 years) corroborates the national data of the study, Health behavior in school-aged children (HBSC) (14.7±1.8 years),^11^ the same is not true for sex. Although both studies present identical percentages, in the present study, the participants were mostly male (n = 210; 53.3%), while in the HBSC, the female participants were predominating (n = 3152; 52.3%).

Since the validity of an instrument is an intrinsic characteristic of it, when applied to a sample, it is possible to perceive that the characteristics of the study population can influence the structure of the instrument. Thus, the exploratory analysis of E3AS pointed to the existence of a pentafactorial construct, and the five factors retained explain 45.3% of the total variance. The performance of reliability and validity studies through exploratory factor analysis indicates that E3AS is a valid and reliable instrument, adapted for the study of adolescent students' attitudes towards sexuality, developed in a school environment and composed of 34 items.

In favor of the conceptual validity of the construct, it should be noted that the factorial structure meets the references of the WHO and the United Nations Children's Fund, the Ministry of Education and the basic principles of logotherapy^8^ ^,^ ^12^ that guide the profile of positive attitudes for adolescent students regarding sexuality, as well as justifying them and giving them meaning. Despite the existence of items that, based on conceptual analysis, would be associated with a certain category, it was found that factor analysis integrated them into different factors. Another argument in favor of the validity of E3AS is related to the fact that it presents good and statistically significant correlations between the factors, which suggests that they evaluate different aspects of the same construct.^13^

In the present work, we suggest evaluating attitudes towards the sexuality of adolescent students through the global score for each factor, obtained by the sum of the scores attributed to each item of that same factor.^10^

The E3AS had good internal consistency, with Cronbach's alpha of 0.766,^9^ and a variation of the corrected correlation values between 0.223 and 0.680, which correspond to good indicators of the total instrument because they are above 0.20.^14^

As for F1, it appears that adolescents recognize family planning as a responsibility that must be shared by both members of the couple. They also consider that places with easy access to health professionals are important, in addition to people they can clarify issues related to sexuality and obtain more information about the prevention of sexually transmitted infections (STIs). These data corroborate the results of several studies regarding the advantage of adolescents having access to “health promotion and education actions, preventing risky sexual behaviors, namely, unplanned pregnancy, STIs, and dating violence”.^15^ This violence, characterized by unequal and unjust gender relations, undermines the healthy development of sexuality and mutual responsibility.^16^ ^,^ ^17^

In F2 of the present study, as in others carried out with adolescents, the reasons evoked for the early initiation of sexual activity are related to consuming alcohol, having an older boyfriend or girlfriend, being afraid that he/she will be angry, or being afraid of abandonment.^15^ ^,^ ^18^ It was concluded that it is mainly the consumption of substances, namely alcohol, that is most associated with the early onset of sexual activity, which may trigger risky sexual behaviors.^19^ It is reinforced that the precocity of the coitarche can have adverse consequences for sexual and reproductive health, such as unintended pregnancy and STIs.^20^

With regard to F3, a higher average value of responses was obtained regarding the following attitudes: asking to respect their decision; in case of pregnancy, asking a health professional for help; and refusing. Corroborating these results, several studies indicate the need to develop personal and socioemotional skills, in order to value equity and diversity and prevent discrimination based on gender, and also the prevention of risky sexual behaviors.^15^ ^,^ ^21^ ^,^ ^22^ In the case of unplanned pregnancies, first-rate structures, such as families, schools, health centers and community resources must assume the responsibility of anticipating, welcoming and guiding adolescents.

In F4, the variables with the highest mean values of attitudes were: clothing defines someone as a man and a woman; each person must identify and accept himself as a man and a woman; and the use of makeup defines someone as a man and a woman.

Taking into account that gender identity refers to the “internal and individual gender experience deeply felt by each person who may or may not live up to social expectations”,^2^ these results confirm the need for demystifying some socially imposed gender roles, to provide real freedom for both sexes, so that women do not feel permanently judged, but also so that men can express their feelings, not assume the dominant role, and refuse intercourse, without their masculinity being called into question.^22^ It is necessary to make men more responsible for reproduction, to educate them on the promotion of human relations of respect for freedom, their own and others, responsibility, coherence, equal rights and pleasure, listening and understanding others.^6^

In F5, the average values for the variable “If a teenager becomes pregnant in a couple, how should they act?” they were, first, keeping the child, followed by having an abortion. Other studies also reveal similar results, concluding that some teenagers' boyfriends blame them for pregnancy, and some suggest termination of the pregnancy. The risks of interpersonal conflicts and the lack of support from parents and boyfriends interfere in adolescents' decision to have an abortion.^21^ ^,^ ^23^ ^,^ ^24^

Using exploratory factor analysis, the instrument consisted of five factors, the value of the total alpha on the 0.766 scale, explaining 45.30% of the total variance, which guarantees the fidelity and validity of the construct.^25^

In relation to sex, and with regard to the different factors, all of them, with the exception of F5, presented statistical significance and higher mean values in females. However, this did not occur in relation to age. Boys, only in F5, showed attitudes with a higher average value and with statistical significance. As for the total, there was also only a statistically significant difference in relation to sex:^26^ adolescents had more limiting attitudes and beliefs than adolescents regarding contraception, dating violence, gender and sexual behavior and in relation to dating.

Given the relevance of this topic in recent years, there has been an increasing interest in assessing the attitude of adolescents with regard to sexuality. This has led to the need to build and adapt a specific instrument for this population. This study allows us to conclude that the instrument presented good reliability and good internal consistency indexes.

Due to the limitation of time, a study of temporal stability was not carried out. It will be left for later studies. Likewise, it was not possible to conduct studies on convergent validity due to the lack of previous studies with similar instruments.

Considering the existence of different contexts in the country and the influence they have, it is suggested that, in future research, confirmatory factor analysis be carried out in larger and more diverse samples of students from other geographic areas.

In conclusion, the psychometric adequacy of E3AS for the Portuguese population indicates that it can be used in future studies in order to assess the attitudes of adolescent students regarding sexuality and contribute to the development of skills, the achievement of goals from Health 2020 and the Ministry of Education.
